# Impact of asymmetrical load magnitude and location on postural sway metrics in healthy young adults

**DOI:** 10.1186/s12998-026-00682-8

**Published:** 2026-10-06

**Authors:** Abdul Rafi, Dean L. Smith, William P. Berg, Mark S. Walsh

**Affiliations:** 1 Department of Kinesiology, Nutrition and Health, Miami University, Oxford, OH USA; 2 Essence of Wellness Chiropractic Center, Eaton, OH USA

**Keywords:** Postural sway, Asymmetrical load carriage, Ergonomics, Shoulder, Hand

## Abstract

**Background:**

Asymmetrical load carriage is a common component of daily activities. Carrying a backpack, handbag, or groceries on one side for example alters the center of mass of the system and may challenge postural stability. Such asymmetrical demands on postural control may be a meaningful stressor to evaluate and address during clinical management.

**Objective:**

To determine the influence of lateral asymmetrical load on postural metrics, whether greater loads produce larger responses, and whether location of the load affects the response.

**Methods:**

Twenty-four healthy young adults (21.13 ± 1.48 years) participated in this repeated-measures study during November 2025. Participants completed five standing conditions including no load, handheld as well as shoulder loads at 15% and 20% body mass using the same backpack. Postural metrics were assessed using a force plate. Outcome measures included average mediolateral center of pressure location (COP X), average anteroposterior center of pressure location (COP Y), 95% ellipse area of postural sway, and total path length. Repeated measures analyses of variance with planned contrasts were performed to evaluate differences among conditions.

**Results:**

Loaded conditions demonstrated greater mediolateral shift in COP X (toward the side of load), greater 95% ellipse sway area, and greater path length compared with the no load condition. Greater magnitude loading (e.g. 20% vs. 15%) amplified both COP X and path length. Hand loading increased mediolateral shift in COP X more than shoulder loading whereas shoulder loading increased 95% sway area more than the hand. Load location did not affect path length. Lateral loading had no statistically significant effect on COP Y.

**Conclusions:**

Both load magnitude and load location influence postural control metrics during asymmetrical load carriage. Clinicians may use this information to guide patients toward safer load carrying strategies when attempting to reduce postural instability and musculoskeletal strain.

## Introduction

Postural stability is a fundamental component of human movement and is essential for maintaining balance during both static and dynamic activities. Even during quiet standing, the body exhibits continuous fluctuations known as postural sway, which reflect the neuromuscular adjustments required to maintain equilibrium [1, 2]. These adjustments can be quantified using center of pressure (COP) measurements obtained from force platforms, providing objective insight into the function of the postural control system. Increased postural sway is commonly interpreted as a reduction in postural stability and may indicate greater demands on neuromuscular control mechanisms [3–5].

Maintenance of balance depends on the integration of visual, vestibular, and somatosensory information by the central nervous system. This sensory information is continuously processed to generate appropriate motor responses that maintain the body’s center of mass within the base of support. When external loads are introduced, compensatory biomechanical and neuromuscular adjustments are required to preserve stability. Consequently, measures of COP behavior provide kinematic and kinetic expressions of the effects of external loading on postural control [4, 6, 7] although they do not directly assess the underlying neural mechanisms.

Load carriage is a common activity in daily life and has been shown to influence postural stability [8]. Previous investigations have demonstrated that external loads alter body alignment, increase muscular demands, and modify postural sway characteristics during standing and locomotor tasks [9]. Research examining symmetrical left/right load carriage, such as backpacks across both shoulders and front-loaded systems, has reported increased COP displacement and greater postural instability as load magnitude increases [7, 10]. These findings suggest that additional external loads place greater demands on the postural control system and require compensatory adaptations to maintain balance.

Although symmetrical loading has been extensively studied, asymmetrical load carriage remains comparatively under-investigated despite its frequent occurrence in everyday life [8]. Individuals routinely carry handbags, grocery bags, briefcases, laptop bags, and occupational equipment on one side of the body. Unlike symmetrical loads, asymmetrical lateral loads shift the center of mass laterally and create frontal-plane moments that challenge balance regulation. To compensate for these perturbations, individuals may alter trunk posture, redistribute weight, and increase activation of stabilizing musculature [11, 12].

Zultowski and Aruin [13] have reported that asymmetrical load carriage influences postural control and have demonstrated effects of both load magnitude and carrying method. However, the relative contributions of load magnitude and load location warrant further investigation. For instance, low body mass load (5%) in the hands does not seem to affect postural sway measures in young adults, but the same load was suggested to have a stabilizing effect in older adults [14]. Load magnitude is an important determinant of biomechanical demand because heavier loads increase the forces acting on the musculoskeletal system and may require larger corrective responses to maintain stability [8, 13]. Studies evaluating progressively heavier loads such as by carrying a backpack on both shoulders [10] or holding a grocery bag in one hand [15] have demonstrated increases in postural sway and COP displacement during standing tasks.

Load location may be equally important. Carrying a load in one hand and carrying the same load using a single-strap shoulder bag create distinct mechanical demands due to differences in load position relative to the body’s center of mass [11]. A handheld load is positioned farther from the trunk and may generate greater frontal-plane torque, whereas a shoulder-supported load is positioned closer to the trunk but may alter upper-body alignment and postural strategy differently [11, 16]. Additionally, a handheld load would decrease the height of the center of mass of the system and a shoulder-supported load would raise it [8], also potentially altering alignment and postural strategy. Despite the practical relevance of these carrying methods, the distinct effects of handheld vs. shoulder supported load placement have not been fully isolated and reported in current research.

Understanding the effects of asymmetrical load carriage has important clinical and ergonomic implications. Manual therapists, chiropractors, rehabilitation professionals, and ergonomists frequently encounter individuals who routinely carry unilateral loads as part of occupational, educational, or daily activities. Repeated exposure to asymmetrical loading may increase postural demands and contribute to musculoskeletal stress, fatigue, and altered movement patterns over time [15, 17–19]. Improved understanding of how load magnitude and carrying configuration influence postural control may contribute to evidence-based recommendations aimed at reducing unnecessary biomechanical stress and promoting safer load carriage practices.

Therefore, the purpose of this study was to examine the effects of asymmetrical load magnitude and load configuration on postural sway in healthy young adults during quiet standing. Specifically, this study used the same backpack apparatus to compare handheld and single-strap shoulder load carriage at loads equivalent to 15% and 20% of body mass. Three hypotheses were tested: (1) the presence of an asymmetrical load would increase postural sway compared with no load; (2) greater load magnitudes would produce greater postural sway responses; and (3) postural sway would differ between handheld and shoulder-supported carrying configurations.

## Methods

### Study design

This laboratory based, repeated-measures experimental study examined the effects of asymmetrical load magnitude and carrying configuration on force platform measures of postural sway during quiet standing. Each participant completed all five experimental conditions, allowing within-subject comparisons across loading configurations and magnitudes. Data collection was conducted in November 2025 in the Biomechanics Laboratory within the Department of Kinesiology, Nutrition and Health at Miami University. The study protocol was approved by the Miami University Institutional Review Board (Protocol #02378r), and all participants provided written informed consent prior to participation.

The repeated-measures design was used in this study to control for the potential influence of individual differences. We estimated sample size in GPower 3.1.9.7 using a moderate to large effect size of 0.08 (e.g. small effect = 0.01; large effect = 0.14) for partial eta square derived from a previous study with similar load [7]. It was determined that 19 participants would provide at least 80% power to detect differences between the 5 conditions at 0.05 level of significance.

### Participants

Participants were recruited through classroom announcements in undergraduate and graduate courses offered by the Department of Kinesiology, Nutrition and Health and through study flyers posted on departmental bulletin boards and in hallways at Miami University. Inclusion criteria consisted of being between 18 and 25 years of age, free from neurological, musculoskeletal, or vestibular disorders affecting balance, and able to safely perform the testing procedures. Exclusion criteria included current lower-extremity, back, or shoulder pain, recent surgery, implanted medical devices, use of medications affecting balance, or any medical condition limiting safe participation. Participants completed a medical screening questionnaire prior to enrollment to confirm eligibility. During screening, participants verbally reported their dominant hand; all enrolled participants identified as right-hand dominant.

### Experimental procedures

Participants wore their usual athletic footwear and stood comfortably on a force plate with their feet centered and shoulder-width apart. Centered referred to a standardized position on the plate that was defined as having the medial malleoli of the left and right feet along the horizontal gridline of the plate and feet equal distance from the plate’s midline [2, 20]. Athletic footwear was permitted to enhance ecological validity, as backpacks are typically used while wearing shoes; this decision was also informed by previous research in university aged adults demonstrating statistically similar postural sway measures during standing performed with and without shoes [2]. A visual target was placed at eye level, 1.5 m away on a wall directly in front of the participant, and participants were instructed to maintain visual fixation on the target throughout testing. During each trial, participants were instructed to stand as still as possible while maintaining an upright posture.

Each participant completed five experimental conditions:

1. No load (NL)
2. Handheld load equivalent to 15% body mass (H15)
3. Handheld load equivalent to 20% body mass (H20)
4. Shoulder load equivalent to 15% body mass (SH15)
5. Shoulder load equivalent to 20% body mass (SH20)

The 15% and 20% body mass conditions were selected because similar backpack loads have been used in previous studies [13, 21, 22]. Further, cross-sectional studies indicate that many students carry backpacks exceeding 10% of their body mass [23, 24]. These two levels allowed us to examine potential dose response effects.

**Fig. 1:**
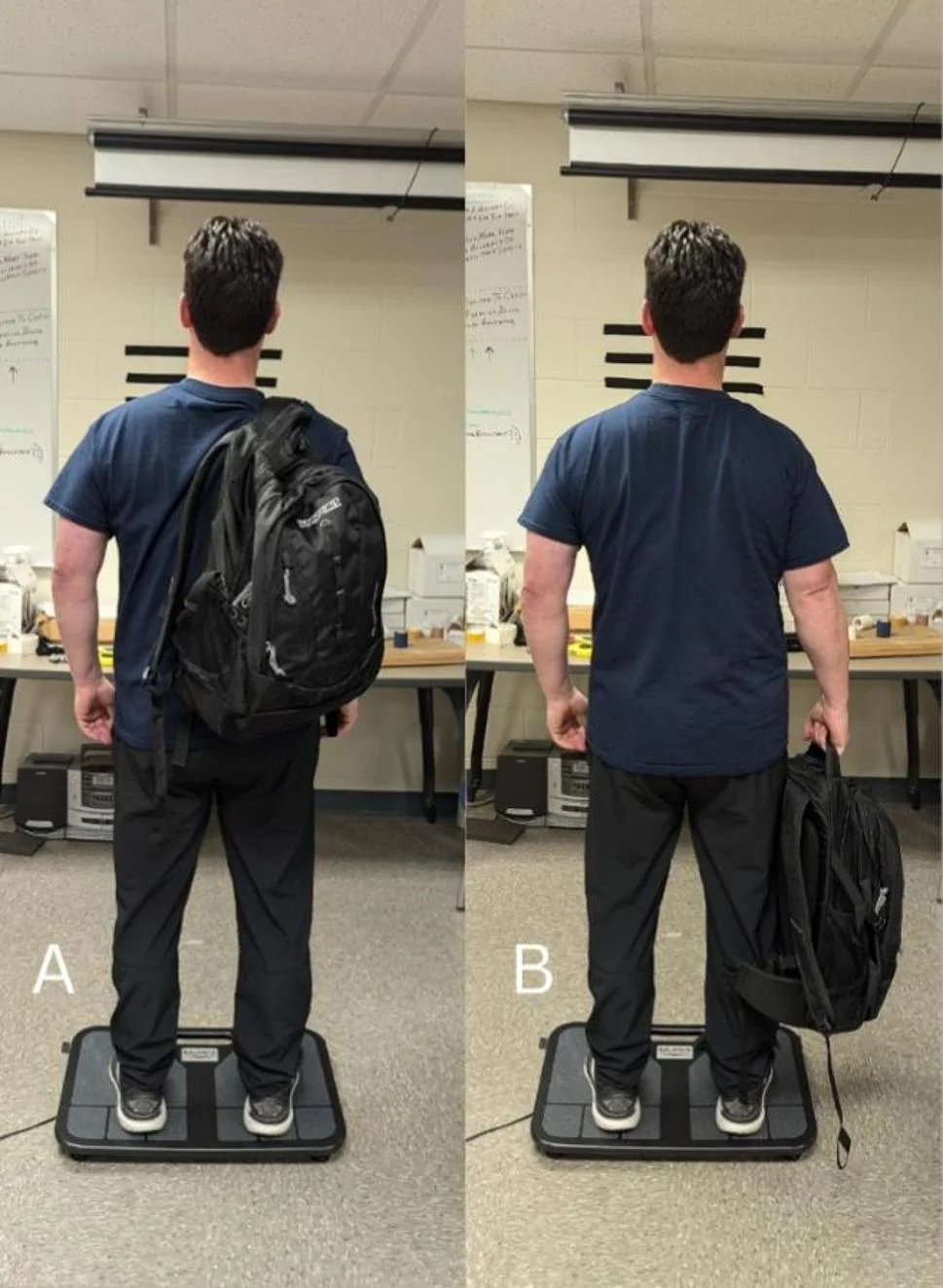
Backpack locations on the shoulder (**A**) and in the hand (**B**)

Participants completed a no load condition and four loaded conditions. For the loaded conditions, participants carried a JanSport Odyssey 39-L backpack loaded with a mass equivalent to 15% or 20% of body mass. Two identical backpacks were prepared for each participant: one loaded to 15% and the other to 20% of body mass. Backpack loads were established using weighted balls (D-Ball.com; 0.45–3.2 kg) combined to achieve the target load within ± 0.1 kg and secured within the backpack to prevent shifting during testing. Mean loads for the 15% and 20% body mass conditions were 10.52 ± 1.92 kg and 14.02 ± 2.56 kg, respectively. The backpack mass (1.3 kg) was not included in the load calculation; however, the same backpack model was used for all participants and conditions. During the handheld conditions, participants carried the backpack unilaterally in their dominant hand using the top grab handle, with the arm at the side. During the shoulder load conditions, participants carried the same backpack over the dominant shoulder using a single shoulder strap (Fig. 1).

Participants completed two 30-second trials for each condition, resulting in a total of 10 recorded trials. Three pseudorandom orders of conditions were implemented between participants to minimize the potential of order and carryover effects. Rest was available between trials and conditions as needed. In practice, most participants took only the few seconds required to transition to and prepare for the subsequent trial; no participant requested an extended rest period. Total testing time was approximately 30 min per participant.

### Postural sway assessment using a force plate

Participants performed quiet bipedal standing on a Balance Tracking System force plate (BTrackS™, San Diego, CA) while wearing the backpack on the shoulder or carrying it in the hand, with eyes open throughout testing. Force plate data were acquired using the Assess Balance software application (Balance Tracking Systems, version 8.5.2) at a sampling frequency of 25 Hz. The data were filtered using a second-order digital Butterworth low-pass filter with a 4 Hz cutoff frequency, implemented point by point in Labview.

### Outcome measures

Postural metrics were assessed using force plate-derived COP measurements. The mean value of the 2 trials per condition was entered into the analysis. Four dependent variables were analyzed:

- Mediolateral (ML) Center of Pressure Displacement (COP X) - average location of the COP relative to the midline of the plate (+ Right, - Left) in centimeters (cm).
- Anteroposterior (AP) Center of Pressure Displacement (COP Y) - average location of the COP relative to the midline of the plate (+ Front, - Back) in cm.
- 95% Ellipse Area (A) - the area of the two-dimensional 95% ellipse derived from the COP data during each trial was used to quantify COP sway area (cm²). The ellipse axes were derived from the eigenvalues of the COP covariance matrix and scaled by 2.45. Area was calculated as A = π*ab*, where *a* and *b* are the semimajor and semiminor axes of the scaled ellipse.
- Total path length (cm) - the cumulative distance traveled by the COP throughout the trial and reflected the amount of postural corrective activity required to maintain balance.

### Statistical analysis

For each selected outcome, a one-way repeated measures ANOVA was used to assess the five conditions (no load, H15, H20, SH15, SH20). Model assumptions of residual normality and variance homogeneity were assessed and verified. When violations of sphericity were detected, Greenhouse-Geisser corrections were applied. All ANOVA tests were performed at α = 0.05. Three, a priori planned, custom contrasts within the ANOVA model were used to evaluate the study hypotheses: (1) load presence will influence postural metrics (no load vs. average of SH15 and H15); (2) greater loads will produce greater responses (average of SH15 and H15 vs. average of SH20 and H20); and (3) there will be a difference between load locations (average of SH20 and SH15 vs. average of H20 and H15). Post hoc tests were not performed as the planned contrasts addressed the study hypotheses. A Bonferroni-adjusted alpha of 0.017 (0.05/3) was used for these 3 planned contrasts within each dependent variable. Statistical analyses were performed using IBM SPSS Statistics (Version 30). Data visualization was performed using R (version 4.6.0; R Core Team, 2023).

## Results

Twenty-four right-handed, healthy young adults, both men (*n* = 10) and women (*n* = 14), completed all testing procedures and were included in the final analysis. The mean age of participants was 21.13 ± 1.48 years, with a mean height of 171.28 ± 9.53 cm and body mass of 70.14 ± 12.79 kg. Complete data were obtained from all 24 participants across all five conditions, and no participants were excluded.

**Fig. 2:**
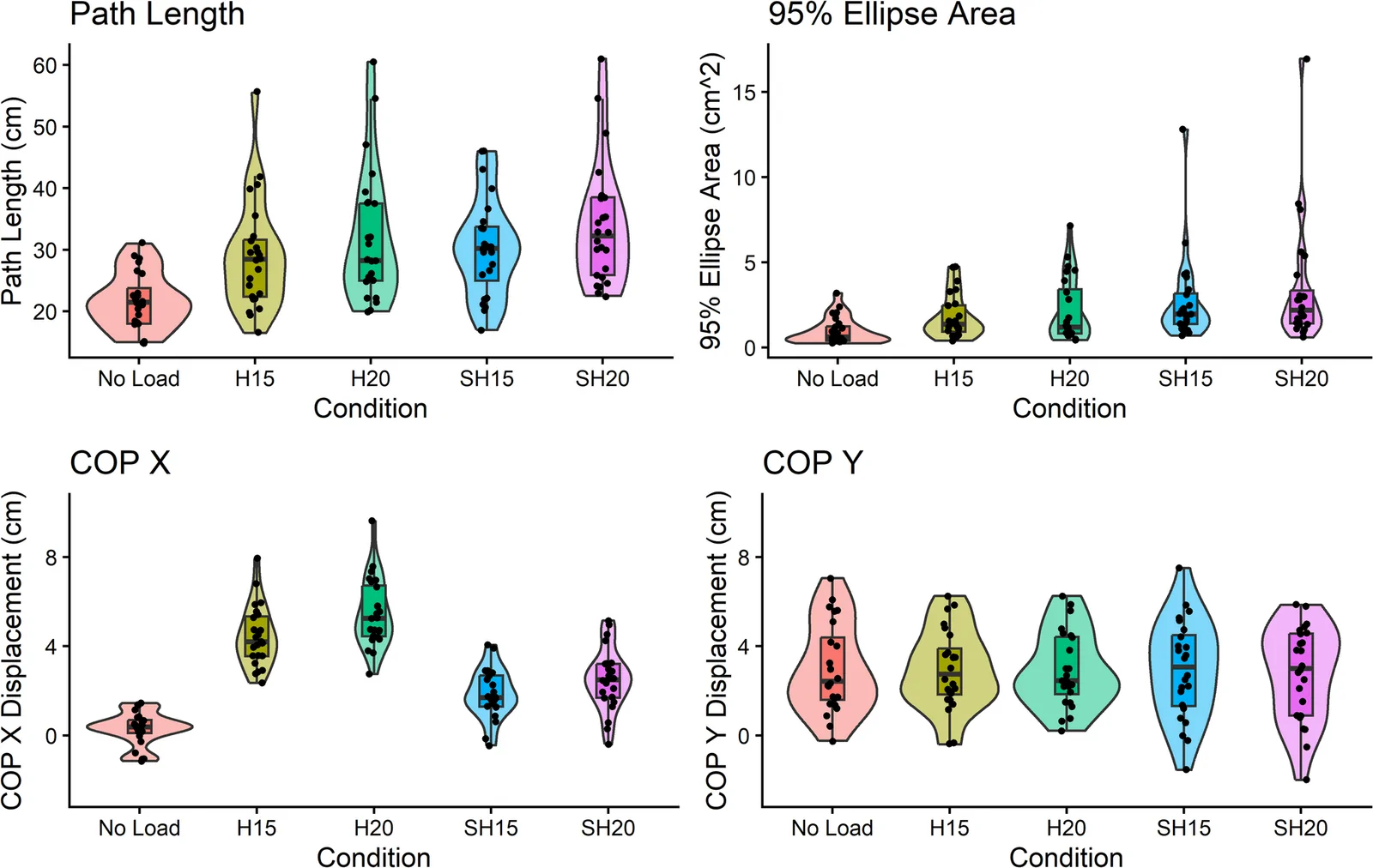
Violin plots showing the distribution of each dependent variable across experimental conditions. Legend: Boxplots show the median and interquartile range. Dots represent individual participants and are displayed with horizontal jitter to reduce overlap and visualize all data points

Figure 2 displays violin plots while Table 1 provides means and standard deviations for each condition and postural metric. Overall, loaded conditions demonstrated greater COP X displacement (toward the side of load), 95% ellipse area, and total path length compared with the no-load condition. In contrast, COP Y remained relatively unchanged across conditions.

**Table 1:** Postural sway variables across experimental conditions (Mean ± SD)

Variable	NL	H15	H20	SH15	SH20
COP X (cm)	0.29 ± 0.74	4.44 ± 1.35	5.48 ± 1.53	1.87 ± 1.10	2.50 ± 1.37
COP Y (cm)	3.00 ± 1.99	2.94 ± 1.80	2.92 ± 1.69	2.95 ± 2.21	2.78 ± 2.12
95% EA (cm²)	0.99 ± 0.78	1.82 ± 1.29	2.17 ± 1.88	2.70 ± 2.54	3.42 ± 3.58
PL (cm)	21.69 ± 4.55	28.96 ± 8.85	32.00 ± 10.77	30.29 ± 8.00	33.85 ± 9.99

*95% EA = 95% Ellipse Area; PL = Path Length; NL = no load; H15 = handheld load at 15% body mass; H20 = handheld load at 20% body mass; SH15 = shoulder load at 15% body mass; SH20 = shoulder load at 20% body mass

### Mediolateral center of pressure displacement (COP X)

A significant main effect of load condition was observed for average location of COP X following Greenhouse–Geisser correction, F(2.55, 58.60) = 145.00, *p* < 0.001, η² = 0.863. The magnitude of this effect indicated that load condition accounted for a substantial proportion of the variance in COP X.

Contrast results are presented in Table 2. All 3 contrasts for COP X were statistically significant, showing that load (e.g. 0% vs 15%) compared to no load increased COP X toward the side of load. Greater loads (e.g. 20% vs 15%) increased COP shift further and, carrying a load in the hand increased COP X more than shoulder loads.

**Table 2:** Planned contrasts for each dependent variable

Outcome	Contrast	MD	95% CI	*p*	η^2^	Interpretation
COP X (cm)	0% vs 15%	-2.86	-3.44, -2.27	< 0.001	0.87	Load increases COP X
	15% vs 20%	-0.84	-1.14, -0.54	< 0.001	0.69	Greater loads increase COP X further
	SH vs H	-2.78	-3.35, -2.20	< 0.001	0.87	Greater COP X with hand loading
COP Y (cm)	0% vs 15%	0.06	-0.42, 0.53	0.76	< 0.005	No evidence of an effect of load amount or location on COP Y
	15% vs 20%	0.10	-0.13, 0.32	0.27	0.05	
	SH vs H	-0.07	-0.67, 0.53	0.77	< 0.005	
PL (cm)	0% vs 15%	-7.93	-11.00, -4.88	< 0.001	0.66	Load increases PL
	15% vs 20%	-3.30	-5.45, -1.15	< 0.001	0.41	Greater loads increase PL further
	SH vs H	1.59	-1.33, 4.52	0.17	0.08	No load location effect on PL

*PL = Path Length; MD = Mean difference. Note: 95% Ellipse Area non-parametric test results are described in the text

### Anteroposterior center of pressure displacement (COP Y)

No significant main effect of load condition was identified for COP Y displacement after Greenhouse–Geisser correction, F(2.43, 55.90) = 0.28, *p* = 0.798, η² = 0.012.

### 95% ellipse area

A Friedman test was conducted because the assumption of normality for the repeated-measures ANOVA was not met. Planned contrasts were performed using Wilcoxon signed-rank tests with appropriate adjustments for multiple comparisons. A significant effect of condition was found, χ2(4) = 50.96, *p* < 0.0001.

Wilcoxon signed-rank tests revealed statistically significant differences with two contrasts. Ellipse area for load at 15% body mass (median [Mdn] = 1.71) was greater than no load (Mdn = 0.63; *p* < 0.001). Carrying loads on the shoulder (Mdn = 2.05) produced greater ellipse area compared to those in the hand (Mdn = 1.29; *p* < 0.001). There was no significant difference between the 15% and 20% load conditions for sway area (*p* = 0.064).

### Total path length

A significant main effect of load condition was observed for total path length, F(3.01, 69.30) = 22.60, *p* < 0.0001, η² = 0.495. Participants exhibited significantly greater path lengths during all loaded conditions compared with the no-load condition, suggesting increased postural corrective activity during asymmetrical load carriage.

Contrast results (Table 2) demonstrated that two contrasts were statistically significant. Load (e.g. 15% vs 0%) compared to no load increased COP path length. Greater loads (e.g. 20% vs 15%) further increased COP path length. However, carrying location was insignificant.

## Discussion

The purpose of this study was to examine the effects of asymmetrical load magnitude and load configuration on postural metrics in healthy young adults during quiet standing. Use of a single backpack system across all loading conditions strengthened the experimental design by enabling load location to be varied while minimizing differences attributable to the carrying equipment. Planned contrasts showed that, relative to the unloaded condition, asymmetrical loading increased COP X, 95% ellipse area, and path length. In contrast, COP Y was unaffected by asymmetrical loading, load magnitude, and carrying configuration. Increasing load magnitude further increased COP X displacement and path length but did not affect 95% ellipse area. Carrying configuration also influenced specific postural control outcomes: handheld loads produced greater COP X than carrying loads on the shoulder, whereas carrying loads on the shoulder produced larger 95% ellipse areas. These findings support the hypothesis that asymmetrical loading challenges postural stability and demonstrate that load magnitude and carrying configuration influence balance through different mechanisms.

### Effects of asymmetrical loading on postural control

The first hypothesis was supported, as asymmetrical loading significantly increased COP X, sway area, and path length compared with the no-load condition. These findings are consistent with previous investigations demonstrating that external loads alter postural control by shifting the body’s center of mass and increasing the neuromuscular demands required to maintain equilibrium [10, 13, 15]. Because the loads in the present study were applied unilaterally, participants were required to compensate for a lateral displacement of the center of mass, resulting in increased postural adjustments during quiet standing.

The observed increases in path length suggest that participants performed a greater number of corrective postural actions when carrying asymmetrical loads. Path length represents the cumulative distance traveled by the COP and is commonly interpreted as an indicator of the amount of neuromuscular activity required to maintain balance. The greater path lengths observed under loaded conditions indicate that participants relied on more active postural regulation to maintain stability when exposed to unilateral loading.

### Influence of load magnitude

The second hypothesis was also supported. Increasing load magnitude from 15% to 20% body mass produced significantly greater COP X and path length. These findings agree with previous research indicating that heavier external loads impose greater biomechanical demands and require larger postural corrections to maintain equilibrium [10, 15]. As load magnitude increases, the lateral displacement of the center of mass becomes greater, increasing the torque that must be resisted by the trunk and lower-extremity musculature.

Interestingly, load magnitude did not significantly influence 95% ellipse area. This finding suggests that while heavier loads increased the amount of corrective activity and lateral COP displacement, they did not substantially expand the overall spatial boundaries of postural sway. One possible explanation is that participants adopted effective compensatory strategies that limited overall sway area despite the increased mechanical demands associated with heavier loads. Healthy young adults may possess sufficient neuromuscular capacity to maintain overall balance performance even when corrective activity increases.

### Influence of carrying configuration

Load carrying location influenced individual postural metrics in different ways. Handheld loads produced significantly greater COP X than shoulder-supported loads, whereas shoulder-supported loads produced significantly greater sway area. These findings suggest that load location influences balance control through multiple mechanisms and that no single postural measure fully captures the effects of asymmetrical loading.

The greater COP X observed during handheld load carriage may be explained by the increased moment arm associated with carrying a load at the side of the body. A handheld load is positioned farther from the body’s center of mass and lower relative to the trunk, increasing frontal-plane torque and requiring larger lateral adjustments to maintain balance. Similar findings have been reported in studies examining unilateral load carriage, where loads positioned farther from the body’s center of mass resulted in larger COP shifts and increased demands on postural control mechanisms [13, 15].

In contrast, shoulder-supported loads resulted in larger sway areas despite producing smaller average COP X. A shoulder bag positions the load closer to the body’s center of mass, potentially reducing the need for large lateral weight shifts. However, the elevated position of the load may require more complex postural adjustments involving the trunk, shoulders, and cervical region. These adaptations may increase the overall spatial variability of COP movement, resulting in a larger sway area despite smaller average mediolateral displacements. Previous investigations examining shoulder bag carriage have similarly reported alterations in posture and balance control associated with asymmetrical upper body loading [11, 16].

Importantly, carrying configuration did not significantly influence path length. Although handheld and shoulder-supported loads affected the direction and spatial characteristics of sway differently, the overall amount of corrective activity required to maintain balance was similar between configurations. This finding suggests that healthy young adults can effectively adapt to different carrying methods, although the strategies used to maintain stability may differ.

### Anteroposterior average location remained unchanged

No significant differences were observed for average location of the COP relative to the midline of the plate in the anteroposterior direction across any experimental conditions. This finding indicates that asymmetrical loading primarily challenged balance in the frontal plane rather than the sagittal plane. The load during the handheld conditions was positioned lateral to the body’s midline and the load during the shoulder conditions was posterolateral. Because all loads were positioned laterally relative to the body’s midline, the primary perturbation occurred in the mediolateral direction. Consequently, although the shoulder condition included a posterior load component, the lack of significant differences in COP Y suggests limited influence in this direction. In contrast, the lateral placement of all loads was associated with changes in COP X behavior, indicating that the loading conditions predominantly influenced postural control in the mediolateral direction.

This observation is consistent with previous studies reporting that the direction of external loading influences the direction of postural adaptation [10, 11, 13]. The absence of significant changes in COP Y suggests that healthy young adults were able to maintain anteroposterior COP average location despite the additional demands imposed by asymmetrical loading.

### Clinical implications

The findings of this study have relevance for clinicians, chiropractors, manual therapists, rehabilitation professionals, and ergonomists. Individuals frequently carry asymmetrical loads during occupational, educational, and daily activities. Repeated exposure to unilateral loading may increase demands on the postural control system and contribute to cumulative musculoskeletal stress over time.

The present findings suggest that both load magnitude and carrying method should be considered when providing ergonomic recommendations. Handheld loads appear to produce greater COP X, whereas shoulder supported loads may increase overall postural variability.

The observed effects of asymmetrical load carriage on ML postural control may be clinically important because ML sway is associated with balance performance and fall related outcomes. Poor balance is a prominent risk factor for falls among older adults [25, 26], and force-platform measures of ML COP movement, particularly average amount, velocity, range, and variability during standing, may help identify older adults at risk of future or repeated falls [27, 28]. ML COP measures also demonstrate associations with age, physical performance, and cognitive function in healthy adults [29].

Given the importance of ML postural control, the observed alterations in COP behavior during asymmetrical load carriage may be particularly relevant for individuals with balance deficits, neurological conditions, or musculoskeletal pain. Asymmetric loads create disproportionately high mechanical and neuromuscular demands, and these effects may be amplified in people with chronic low back pain [30]. Clinicians should therefore consider evaluating load carriage habits when assessing patients who report balance difficulties or discomfort associated with carrying bags, equipment, or occupational tools.

Potential clinical applications include screening postural control responses to asymmetrical loading, incorporating loaded and task specific activities into balance assessment, identifying load related symptom provocation and compensatory movement patterns, educating patients regarding load carriage management strategies, and prescribing progressive balance and trunk motor control interventions under asymmetrical loading conditions. These considerations may also inform return to work and return to activity decisions by evaluating an individual’s capacity to tolerate the loading demands of daily, occupational, and recreational tasks.

### Limitations and future directions

Several limitations should be acknowledged. First, the sample consisted exclusively of healthy young adults recruited from a university population, limiting the generalizability of the findings to older adults and clinical populations. Second, postural sway was assessed only during quiet standing. Dynamic tasks such as walking, stair negotiation, and occupational activities may produce different responses to asymmetrical loading. Third, the study focused on immediate postural responses and did not evaluate the long-term consequences of repeated asymmetrical load carriage.

While it is possible that males and females may respond differently to the loading conditions, the present study was not designed or sufficiently powered to evaluate sex differences or sex by condition interactions. Given the relatively small sample size, we focused our analyses on the primary within subject effects of load amount and load location. We do think that future studies with larger samples may be warranted to examine potential sex specific responses.

Future investigations should examine clinical populations, evaluate dynamic movement tasks, and explore neuromuscular activation patterns associated with different carrying configurations. Further research may also investigate the cumulative effects of prolonged asymmetrical loading on spinal mechanics, musculoskeletal health, and injury risk. The results of this study suggest unilateral loading represents a functional challenge to the neuromuscular system. Clinicians and exercise specialists may use this data to incorporate into prevention programs as well as rehabilitation management depending on the needs and goals of the individual.

## Conclusion

Asymmetrical load carriage significantly altered postural control in healthy young adults. The presence of a unilateral load increased the shift of average COP X, sway area, and path length, while greater load magnitudes further increased COP X and corrective activity. Handheld loads produced greater COP X, whereas shoulder-supported loads resulted in greater sway area. No significant shift of COP Y was observed with lateral loading. These findings demonstrate that both load magnitude and carrying configuration influence postural control performance and highlight the importance of considering load carriage practices when developing ergonomic and clinical recommendations.

## Data Availability

The datasets used and/or analyzed during the current study are available from the corresponding author on reasonable request.
